# Bluetongue Serotype 3 in Israel 2013–2018: Clinical Manifestations of the Disease and Molecular Characterization of Israeli Strains

**DOI:** 10.3389/fvets.2020.00112

**Published:** 2020-03-06

**Authors:** Natalia Golender, Velizar Bumbarov, Avi Eldar, Alessio Lorusso, Gabriel Kenigswald, Joseph Seffi Varsano, Dan David, Shani Schainin, Ilan Dagoni, Iosef Gur, Alon Kaplan, Anna Gorohov, Ori Koren, Eldad Oron, Yevgeny Khinich, Ily Sclamovich, Abraham Meir, Giovanni Savini

**Affiliations:** ^1^Department of Virology, Kimron Veterinary Institute, Bet Dagan, Israel; ^2^OIE Reference Laboratory for Bluetongue, Istituto Zooprofilattico Sperimentale dell'Abruzzo e del Molise (IZSAM), Campo Boario, Teramo, Italy; ^3^Hachaklait Veterinary Services, Caesarea, Israel; ^4^Indi-Vet Ltd., Ashdod, Israel

**Keywords:** orbivirus, sheep, cattle, clinical signs, phylogeny, descriptive epidemiology

## Abstract

In this paper, the results of the diagnostic activities on Bluetongue virus serotype 3 (BTV-3) conducted at Kimron Veterinary Institute (Beit Dagan, Israel) between 2013 and 2018 are reported. Bluetongue virus is the causative agent of bluetongue (BT), a disease of ruminants, mostly transmitted by competent *Culicoides* species. In Israel, BTV-3 circulation was first detected in 2013 from a sheep showing classical BT clinical signs. It was also evidenced in 2016, and, since then, it has been regularly detected in Israeli livestock. Between 2013 and 2017, BTV-3 outbreaks were limited in sheep flocks located in the southern area only. In 2018, BTV-3 was instead found in the Israeli coastal area being one of the dominant BTV serotypes isolated from symptomatic sheep, cattle and goats. In Israeli sheep, BTV-3 was able to cause BT classical clinical manifestations and fatalities, while in cattle and goats infection ranged from asymptomatic forms to death cases, depending on either general welfare of the herds or on the occurrence of viral and bacterial co-infections. Three different BTV-3 strains were identified in Israel between 2013 and 2018: ISR-2019/13 isolated in 2013, ISR-2153/16 and ISR-2262/2/16 isolated in 2016. Sequencing and phylogenetic analysis of these strains showed more than 99% identity by segment (Seg) 2, 5, 6, 7, and 8 sequences. In contrast, a wide range of diversity among these strains was exhibited in other viral gene segments, implying the occurrence of genome reassortment between these local circulating strains and those originating from Africa. The genome sequences of the BTV-3 isolated in 2017 and 2018 were most closely related to those of the ISR-2153/16 strain suggesting their common ancestor. Comparison of BTV-3 Israeli strains with those recently detected in the Mediterranean region uncovered high percentage identity (98.19–98.28%) only between Seg-2 of all Israeli strains and the BTV-3 Zarzis/TUN2016 strain. A 98.93% identity was also observed between Seg-4 sequences of ISR-2019/13 and the BTV-3 Zarzis/TUN2016 strain. This study demonstrated that BTV-3 has been circulating in the Mediterranean region at least since 2013, but, unlike the other Mediterranean strains, Israeli BTV-3 were able to cause clinical signs also in cattle.

## Introduction

Bluetongue (BT) is a non-contagious, arthropod-borne viral disease of domestic and wild ruminants, listed as a notifiable disease by the World Organization for Animal Health (OIE). The Bluetongue virus (BTV) is the prototype member of the *Orbivirus* genus within the *Reoviridae* family (1, 2).

BTV is a double-stranded (ds) RNA virus (3), its genome consists of ten segments (Seg-1 to Seg-10) of linear dsRNA coding 7 structural (VP1–VP7) and 5 non-structural (NS1, NS2, NS3/NS3a, NS4, and NS5) proteins (4) At present twenty-eighth distinct BTV serotypes have been officially recognized based on Seg-2 gene sequence (5, 6). Other putative novel BTV serotypes have also been described (7–11).

Transmission between mammalian hosts and spread of the infection rely mostly on competent *Culicoides* species (12–14), so the presence of the disease is then strictly related to the distribution of competent vectors (2). Even though they don't seem to be epidemiologically important, vertical and horizontal transmissions have also been described (15–18). For BTV-26, BTV-27 v02 and, probably, BTV-X ITL2015 and BTV-28 transmission by direct contact has been demonstrated or hypothesized (6, 10, 19, 20).

Clinical signs of BT are more severe and most commonly observed in sheep or in white-tailed deer, often leading to animal fatality especially in naïve animals (1, 2). In cattle, BTV infection is usually asymptomatic, although symptoms were reported after infection with some strains (21–24).

As a RNA virus with a segmented genome, BTV can undergo reassortment which can occur when a cell is simultaneously infected with more than one BTV strain and involves the packaging, into a single virion, of full length of genomic segments of different ancestry. Reassortment in BTV is very flexible, and can involve any genome segment (25–27). However, the genome sequence of BTV isolates generally reflects their geographic origins (28–30).

Seg-2 and Seg-6 encoded the BTV outer capsid proteins VP2 and VP5. They represent the primary target for neutralizing antibodies generated during infection of the mammalian host (31–33). Their highly variable sequences are associated with virus serotype (particularly in VP2/segment) (34–36) and, within each serotype, with the geographic origin of the virus strain (28, 34–37).

Among BTV serotypes, Seg-2/VP2 sequences of BTV-3, BTV-16, and BTV-13 are closely related. All of them are included within the nucleotype B reflecting a serological relationship (3). BTV-3 strains can be subdivided into at least two main clusters. 1st cluster includes strains originated from Africa, Mediterranean Basin and North America (western topotypes, w); 2nd-includes strains originated from Japan, India, and Australia (eastern topotypes, e). The nucleotide (nt) identity between Western and Eastern topotype Seg-2 can be as small as 71.5% (7, 38–43).

Between 2016 and 2018, two novel BTV-3 western strains have been identified in two different geographical areas of Tunisia-one in the north-eastern part of the country (Peninsula of Cap Bon, prototype BTV-3 TUN2016) and the other in the South-East near by the border with Libya (prototype strain BTV-3 TUN2016/Zarzis). The BTV-3 TUN2016 spread in 2017 to Italy infecting a single 3-year-old female crossbred sheep belonging to a flock located in the municipality of Trapani, Sicily, which are 150 km distant from Peninsula of Cap Bon (7, 39, 40) and in 2018 in the Southern area of Sardinia causing numerous outbreaks (38). Clinical signs in infected sheep included depression, fever, nasal discharge, submandibular edema, and crusted discharge around the nostrils. Four animals died because of the severity of infection (38). In 2016, another BTV-3 strain closely related to TUN2016/Zarzis strain was detected in Egypt (38).

The present paper reports the results of the diagnostic activities on BTV conducted at Kimron Veterinary Institute between 2013 and 2018 and the evidence of BTV-3 circulation in Israel. Clinical signs of infected sheep, goats and cattle along with the genetic characterization and phylogenetic analysis of the BTV-3 strains are also described.

## Materials and Methods

### Field Samples

During years 2013, 2016, 2017, and 2018, 3,149 samples (714 in 2013, 669 in 2016, 744 in 2017, and 1,022 in 2018) from domestic and wild ruminants were collected and examined at the Kimron Institute, Beit Dagan, Israel (KVI). Samples included whole blood from symptomatic animals, spleen or/and lung samples from dead animals and spleen, lung, placenta and brain samples from aborted fetuses. Details on number, samples, species from which samples were collected are shown in Table 1.

**Table 1:** Field samples tested by Pan-BTV RT-qPCR from different kinds of domestic and wild/zoo ill or dead animals and subsequent virus isolations during 2013, and 2016–2018.

**Species**		**Cattle**			**Sheep**			**Goat**			**Wild ruminants**				
**Year**		**w.b**.	**s/l**	**a.f**	**w.b**.	**s/l**	**a.f**	**w.b**.	**s**.	**a.f**	**w.b**.	**s**.	**a.f**	**Total samples**	**Total VI**
2013	No. of tested samples	564	10	3	68	28	3	6	12	2	0	16	2	714	
	No. of pos. samples	113	3	1	18	13	2	0	5	1	0	2	0	158	
	No. of isolated BTV-2	1													1
	No. of isolated BTV-3				1										1
	No. of isolated BTV-4	1			6										7
	No. of isolated BTV-16	3			3										6
2016	No. of tested samples	484	41	10	73	20	18	1	4	5	0	11	2	669	
	No. of pos. samples	159	13	1	49	5	0	0	1	0	0	0	0	228	
	No. of isolated BTV-2				3										3
	No. of isolated BTV-3				3										3
	No. of isolated BTV-4	1													1
	No. of isolated BTV-8	24	1		26										51
	No. of isolated BTV-15	1													1
2017	No. of tested samples	402	35	7	173	41	10	9	24	0	17	23	3	744	
	No. of pos. samples	93	6	0	51	9	0	0	6	0	1	2	0	168	
	No. of isolated BTV-2	1													1
	No. of isolated BTV-3				4										4
	No. of isolated BTV-4	1			12										13
	No. of isolated BTV-6	6			10										16
	No. of isolated BTV-15	6													6
2018	No. of tested samples	629	18	53	72	32	156	8	21	14	4	13	3	1,022	
	No. of pos. samples	217	6	1	36	0	6	3	2	0	0	0	0	271	
	No. of isolated BTV-2	1													1
	No. of isolated BTV-3	1			6			1							8
	No. of isolated BTV-4	1			9										10
	No. of isolated BTV-6				2										2
	No. of isolated BTV-15	9													9

*No., number; w.b, whole blood samples; s/l, spleen or lung samples; s., spleen samples; a.f., aborted fetus; VI, virus isolation. Data from 2017 was also used in publication (44)*.

### Clinical and Epidemiological Follow Up

Farms where domestic clinically ill ruminants were confirmed by laboratory tests as BTV-3 infected were recorded and numbered in **Table 3**. Information in **Table 3** included location of the farm or animal grazing place, type of farming, breed, and number of animals in the farm/group. All clinical events observed in the farms were followed and reported (**Table 4**).

### Laboratory Tests

Internal organs were examined for aerobic and anaerobic bacteria and *Salmonella* spp. growth according to standard procedures (45).

From all field samples and virus isolates (chicken embryo homogenate and tissue culture supernatant) viral RNA was extracted using Invisorb Spin Virus RNA Mini Kit (STRATEC Molecular, Berlin, Germany), and by MagMAX™ CORE Nucleic Acid Purification Kit (Thermo Fisher Scientific), according to recommendations of manufacturers. To detect bovine ephemeral fever virus (BEF), the RNA extraction and RT-qPCR were performed on white blood cells from whole EDTA blood samples of cattle origin according to Erster et al. (46).

Presence of Malignant Catarrhal Fever virus (MCFV) DNA was determined according to the method described by Cunha (47). Most samples from cattle were also tested for the presence of Epizootic Hemorrhagic disease virus (EHDV) RNA by EHDV Real-Time PCR Kit (Applied Biosystems™, Thermo Fisher Scientific Inc., Lissieu, France) according to manufacturer's instructions. The method described by Boxus (48) was used to detect Bovine Respiratory Syncytial virus (BRSV) RNA from lung cattle samples, while an in-house RT-qPCR (unpublished) and Kishimoto et al. (49) methods were used for detecting bovine Parainfluenza 3 (BPI3) and bovine Corona virus (BCoV) RNAs, respectively. A specific RT-qPCR was used for detection and identification Simbu serogroup RNA viruses in plasma samples (50) and, finally, the VetMAX™ BTV NS3 All Genotypes Kit (Applied Biosystems™, Thermo Fisher Scientific Inc., Lissieu, France) RT-qPCR kit targeting Seg-10 of the BTV genome was used for detecting BTV RNA.

Those samples which were positive to the BTV RT-qPCR with Cycle Threshold (Ct) values ≤33, were further tested for determining the serotype. Virotype® BTV pan/4 RT-PCR and Virotype® BTV pan/8 RT-PCR kits (QIAGEN, Leipzig, Germany) were applied directly to RNA extracted from diagnostic samples to detect and identify BTV-4 and BTV-8 serotypes, respectively. The same samples were also retroactively tested for the presence of BTV-3 by an in-house specific RT-qPCR according to the method described by Lorusso et al. (7).

In an attempt to isolate BTV, all BTV RT-qPCR positive samples were inoculated in 9 to 11 day old embryonated chicken eggs (ECE) according to the method described by Komarov and Goldsmith (51) and adopted by Golender et al. (52). In the homogenate of the ECE organs, the presence of BTV was confirmed by BTV RT-qPCR. BTV-positive ECE homogenates were subsequently tested for the presence of the RNA of all BTV serotypes that have circulated in Israel and neighboring countries during the recent decades (BTV-2, −3, −4, −5, −8, −12, −15, −16, and−24) by using a conventional specific in-house RT-PCR (primers are listed in Table S1) using a One-Step RT-PCR kit (Qiagen, Hilden, Germany).

Primers 3VP2-1F (5′-GTT AAA AAC GCT GTC CCG AGA-3′) and 3VP2-637R (5′-GAG CGC CCA CTC TAA ATT CCT C-3′) targeting a portion of 658 bp of the Seg-2 of BTV-3 by RT-PCR were used for BTV-3 identification. All positive in in-house conventional serotype specific RT-PCR tested BT virus isolates from all serotypes were consequently sequenced.

All viral-segment amplicons of Israeli BTV-3 were sequenced by standard Sanger methods with an ABI 3730xl DNA Analyzer (Hylabs, Rehovot, Israel). The cDNA fragments were purified with a MEGAquick-spin Total Fragment DNA Purification Kit (iNtRON Biotechnology, Gyeonggi-do, South Korea). The resulting nucleotide sequences were assembled and nucleotide (nt) and amino acid (aa) sequences were aligned and pairwise compared by using Geneious version 9.0.5 (Biomatters, Auckland, New Zealand). Phylogenetic trees were constructed with the Mega 7.1 software (53).

## Results

### BTV Detection by RT-qPCR From Field Samples

Data regarding the presence of BTV in the samples examined by BTV RT-qPCR between 2013 and 2018 are shown in Table 1. A total of 3,149 samples were tested for the presence of BTV. Of these 792 were found positive by BTV RT-qPCR. Of the 714 samples collected in 2013, 125 were found positive for BTV, 228 of the 669 collected in 2016, 168 of the 744 collected in 2017, and 271 of the 1,022 collected in 2018. Details on the BTV results obtained from the tested samples are also shown in Table 1.

### Field Samples Serotyping by Specific RT-qPCR, 2018

Of the 271 field samples collected during 2018 and found positive for BTV by RT-qPCR, 175 (138 from cattle, 32 from sheep and 5 from goats) had Ct values of ≤33. These positive samples were further tested by BTV-4, BTV-8 and BTV-3 specific RT-qPCR. Of the 175 BTV positive samples, 25 were found positive for BTV-3 (15 whole blood samples from 14 cattle; from one cattle blood samples were collected twice), 8 from sheep and 2 from goats) and 32 for BTV-4 (13 from cattle, 18 from sheep and 1 from a goat). Additional 2 samples, one newborn calf and one placenta from case of abortion in sheep, were found positive to in-house conventional RT-PCR unveiling Israeli BTV-4 strain in the both cases. One whole blood sample from sheep and 2 samples from cattle (one whole blood and one spleen samples), were contemporaneously positive for BTV-3 and BTV-4 in specific RT-qPCRs. All samples positive for BTV-4 were received between March and December 2018, while all samples positive for BTV-3 were received between August and December 2018. No BTV-8 positive samples were found in 2018 (Table 2).

**Table 2:** Field samples tested by BTV serotype specific RT-qPCRs from different kinds of domestic ill or dead animals during 2018.

**Species**		**Cattle**		**Sheep**	**Goat**		
**Specific RT-qPCR**		**w. b**.	**s/l**	**w. b**.	**w. b**.	**s**.	**Total**
BTV-3	No. of tested samples	133	5	32	3	2	175
	No. of positive samples	13	2	8	1	1	25
BTV-4	No. of tested samples	133	5	32	3	2	175
	No. of positive samples	11	2	18	1	0	32
BTV-8	No. of tested samples	133	5	32	3	2	175
	No. of positive samples	0	0	0	0	0	0

*No., number; w.b., whole blood samples, s/l, spleen or lung samples; s., spleen samples*.

### Virus Isolation

Of the 792 BTV RT-PCR samples inoculated into ECE, 136 BTV strains were isolated, 15 were isolated from the batch of samples collected in 2013, 59 from the batch of samples collected in 2016, 40 from the samples collected in 2017 and 25 from the samples collected in 2018. The 15 BTV strains isolated in 2013 were identified as BTV-2 (*n* = 1), BTV-3 (*n* = 1), BTV-4 (*n* = 7), and BTV-16 (*n* = 6). The 59 strains isolated in 2016 included BTV-2 (*n* = 3); BTV-3 (*n* = 3), BTV-4 (*n* = 1), BTV-8 (*n* = 51), and BTV-15 (*n* = 1). In one sample BTV-3 and BTV-8 mixed infection was found. The 40 BTV strains isolated in 2017 comprised BTV-2 (*n* = 1), BTV-3 (*n* = 4), BTV-4 (*n* = 13), BTV-6 (*n* = 16), and BTV-15 (*n* = 6) (Table 1). The 30 BTV isolated in 2018 included BTV-2 (*n* = 1), BTV-3 (*n* = 8), BTV-4 (*n* = 10), BTV-6 (*n* = 2), and BTV-15 (*n* = 9). In 4 occasions BTV-3 and BTV-4 mixed infection was observed (Tables 1, 3, 4). Notably, where only one out of four samples was positive in BTV-4 specific RT-qPCR, from which mixed BTV-3 and BTV-4 were isolated, probably pointing out undetected cases by BTV-4 RT-qPCR test.

**Table 3:** Geographic localities and epidemiological aspects on sheep and cattle farms, where BTV-3 was identified.

**Year**	**Locality/geographic zone/distinct**	**Farm**	**Species/No. of animals in the farm**	**Breed**	**Affected group**	**No. of dead animals/No. of ill animals/total animals in affected group**	**Morbidity/case mortality (%)**
2013	Pri Gan/Negev desert/Southern Distinct	1	Sheep/no data	Merino, Romanov, Charolais	No data	No data	No data
2016	Moshav Tkuma/Negev desert/ Southern Distinct	2	Sheep/400	Fin, Merino and Afek (Borulla)	All ages	10/50/400	12.5/20
2016	Mikne Dekel/Negev desert/ Southern Distinct	3	Sheep/1250	Charolais	Adult ewes and 5-month old lambs	60/150/no data	No data/40
2017	Moshav Lachish/ Negev Desert/ Southern Distinct	4	Sheep/750	Merino, Romanov, Asaf, Puld-Dorset	Lambs 4–15 month and primipara ewes	20–30/100/no data	No data/20–30
2017	Sde David/ Negev desert/ Southern distinct	5	Sheep/450	Asaf x Merino	Lambs of different ages	10/50/150	33.3/20
2017	Rahat/Negev desert/Southern distinct	6	Sheep/no data	No data	No data	No data	No data/high
2018	Kfar Silver/Sothern distinct	7	Goat/700	Alpine	Adult doe	1/1/350	0,14/100
2018	Moshav Avigdor/ Southern distinct	8	Cattle/ 550	Holstein-Friesian	Pregnant calf (24 m) and adult cows	4/4/550	0,72/100
2018	Moshav Lachish/ Negev Desert/ Southern Distinct	9	Sheep/ 900	Merino, Romanov, Asaf, Puld-Dorset	Lambs 4-12 month	30-40/200/400	10/15-20
2018	Mikne Dekel/Negev desert/ Southern Distinct	10	Sheep/1250	Charolais	Lambs and adult ewes	5/no data/1250	<1/no data
2018	Moshav Nordia/Sharon plain/ Central distinct	11	Sheep/2200	Asaf	Lambs and adult ewes	No data/no data/2200	No data
2018	Kfar Maimun/ Southern Distinct	12	Goat/400	Saanen	Pregnant does	0/40/150	0/0
2018	Kibbutz Kvutsat Yavne/ Southern coastal plate/Sothern distinct	13	Cattle/760	Holstein-Friesian	2 adult pregnant cows	2/2/400	0.5/100
2018	Moshav Beer Tuvya/ Shfela/ Southern Distinct	14	Cattle/ no data	Holstein-Friesian	Adult bulls	No data	No data
2018	Moshav Nir Galim/ Southern coastal plain/ Southern distinct	15	Cattle/ 800	Holstein-Friesian	4 month old calves	15/20/400	5/75
2018	Kfar Vitkin/ Hefer Valley/ Central distinct	16a	Cattle/185	Holstein-Friesian	18-month old female calf	0/1/30	3.3/0
2018	Kfar Vitkin/ Hefer Valley/ Central distinct	16b	Cattle/400	Holstein-Friesian	18-month old female calf	0/1/180	0.56/0
2018	Havat Shikmim/ Negev desert/Southern distinct	17	Sheep/4000	Charolais	Adult ewes	0/100/2000	5/0
2018	Beit Yitzhak/ Central distinct	18	Cattle/200	Holstein-Friesian	One-year-old female calf an one adult cow	2/2/200	1/100
2018	Kfar Shmuel/ Shfela/ Central distinct	19	Cattle/120	Holstein-Friesian	An adult cow	0/1/70	1.3/0
2018	Kibbutz Berot Ithak/ Central distinct	20	Cattle/574	Holstein-Friesian	A calf and an adult milking cow	0/2/574	0.35/50
2018	Kibbutz Yad Mordechai/Southern Distinct	21	Cattle/1200	Holstein-Friesian	A single one-year-old female calf	0/1/100	1/0

**Table 4:** Clinical signs, virus isolation, and additional laboratory diagnoses found in BTV-3 affected domestic ruminants.

**Farm**	**Beginning of disease**	**Clinical signs**	**Sample date**	**Pos/total**	**BTV-3 strain**	**Other laboratory investigations/method**	**Pos laboratory tests/method**
1	Beg-Nov-2013	Classical clinical manifestations of BT disease	Beg-Nov-2013	2/3	ISR-2019/13	VI	No
2	End-Sep-2016	Fever, nasal discharge, hyperemia and ulcers in the oral and/or nasal mucosa, face and/or thorax edema, fatigue, and apnea	28-Sep-2016	1/1	ISR-2153/16	VI	No
3	Oct-2016	Fever, nasal discharge, severe hyperaemia, ulceration of oral and/or nasal mucosa, severe face and/or thorax edema, recumbency, and apnea	16-Oct-2016	3/3	ISR-2262/2/16	VI	BTV-8/VI from another two samples
4	Mid-Sep-2017	Fever, followed by lameness and stiffness in legs and back muscles, conjunctivitis, nasal discharge, ulceration of oral and nasal mucosa, recumbency, fatigue, mild respiratory distress, and a few abortions	14-Sep-2017; 28-Nov-2017	3/3; 2/2	ISR-2107/2/17; ISR-2396/2/17	VI	BTV-6/VI from another sample
5	Beg-Sep-2017	Fever followed by skin erythema	6-Sep-2017	3/3	ISR-2079/2/17	VI	BTV-6/VI
6	Oct-2017	Fever, fatigue, reluctance to move, inappetence, pneumonia, and subsequent high mortality	16-Oct-2017	1/1	ISR-2219/17	VI	No
7	23-Aug-2018	Abrupt death	23-Aug-2018	1/1	No	BTV-4,−8/PCR; VI	No
8	Beg-Sep-2018	Fever, recumbency with following death	2-Sep-2018	1/1	No	BTV-4,−8/PCR; BEF/PCR; BEF/SN; VI; bacterial culture	*Clostridium perfringens* (calf) and few cases in the farm
9	Beg-Sep-2018	Classical clinical manifestations of BT disease	13-Sep-2018; 29-Oct-2018	2/2	ISR-1965/18; ISR-2210/18	BTV-4,−8/PCR; VI	No
10	Mid-Sep-2018	Fever, facial, neck, and hind limbs edema, stiff gate	17-Sep-2018	3/3	ISR-1975/1/18; ISR-1975/2/18	BTV-4,-8/PCR; VI	BTV-4/PCR; BTV-4/VI
11	Beg-Oct-2018	Fever, lips and facial edema, stiff gate	10-Oct-2018	3/4	ISR-2078/2/18; ISR−2078/4/18	BTV-4,-8/PCR; VI	BTV-4/PCR; BTV-4/VI
12	Oct-2018	Abortions	21-Oct-2018	2/2	ISR-2144/2/18	BTV-4,-8/PCR; VI; pan-Simbu/PCR	BTV-4/PCR (in another goat sample)
13	End-Oct-2018	Recumbency, death	24-Oct-2018	1/1	No	BEF/PCR; BEF/SN; BTV-4,-8/PCR; VI	No
14		No data	24-Oct-2018; 4-Nov-2018; 21-Nov-2018; 19-Dec-2018	1/8; 1/4; 2/10; 1/10	No	BTV-4,-8/PCR; VI	No
15	End-Oct-2018	Recumbency and death after few hours from beginning of illness. Post mortem examination- pneumonia	25-Oct-2018	1/1	No	BTV-4,-8/PCR; VI; BSRV/PCR; BPI-3/PCR; BCoV/PCR; bacterial culture	*Salmonella* spp.
16	End-Oct-2018	Fever, recumbency, tremor/stiffness of neck muscles	25-Oct-2018; 31-Oct-2018	2/2	No	BTV-4,-8/PCR; pan-Simbu/PCR; EHDV/PCR; VI	No
17	Oct-2018	Abortions	28-Oct-2018	2/3	No	BTV-4,-8/PCR; pan-Simbu/PCR; VI	SHUV/PCR
18	Beg-Nov-2018	Hypersalivation, neural ketosis, neck tense muscles (adult cow); fever, fatigue, conjunctival hyperemia (calf)	28-Oct-2018; 5-Nov-2018	1/1	ISR−2255/18	BTV-4,-8/PCR; EHDV/PCR; VI-adult cow; BTV-4,-8/PCR;BEF/PCR; EHDV/PCR; BLV/ID; VI- calf	BEF/PCR (calf)
19	Beg-Nov-2018	Fever, indifference, inapetence, sharp decrease in milk production	4-Nov-2018	1/1	No	BTV-4,-8/PCR; MCFV/PCR VI	No
20	End-Nov-2018	Foamy salivation, nystagmus, hypothermia, mucosal cyanosis and death (calf). Post parturient ketosis, sharp blindness, endometritis, tachycardia (adult cow); after symptomatic treatment- recovery. Generally in farm- cases of diarrhea and abortions	29-Nov-2018; 16-Dec-2018	1/1	No	BTV-4,-8/PCR; VI; MCFV/PCR	BTV-4/PCR (calf)
21	Mid-Dec-2018	Bloody-purulent nasal discharge	13-Dec-2018	1/1	No	BTV-4,-8/PCR; VI, IBRV/PCR; BEF/PCR; EHDV/PCR; IBRV/ELISA-Ab	BTV-4/PCR

*Pos/total, number of positive and number of total samples received from the farm; VI, virus isolation; BEF, bovine ephemeral fever; EHDV, epizootic hemorrhagic disease virus; MCFV, malignant catarrhal fever virus; BLV, bovine leukemia virus, pan-Simbu- panel of Simbu serogroup viruses (Peribuniaviridae family); ID, immune diffusion; BRSV, bovine respiratory syncytial virus; BPI−3, bovine parainfluenza 3 virus; BCoV, bovine corona virus; SHUV, Shuni virus; IBRV, infectious bovine rhinotracheitis virus*.

### Clinical and Epidemiological Follow Up Associated With BTV-3 Infection

**Figure 1:**
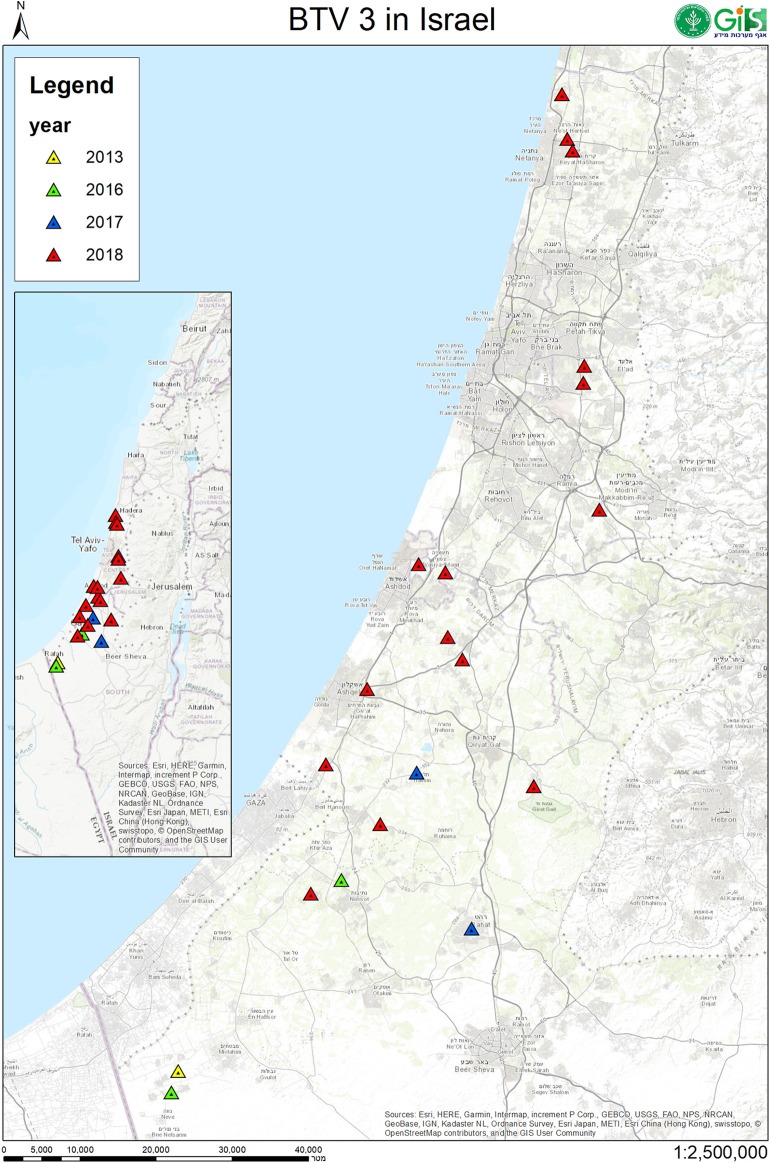
Geographic location of Israeli sheep, goat and cattle farms where BTV-3 was identified. Location of farms with BTV-3 affected domestic ruminants in 2013 were sign by yellow, 2016- by green, 2017- by blue and in 2018 by red triangles.

In this study, BTV-3 was detected in 20 farms numbered from 1 to 21 (Tables 3, 4). From 2013 till 2017, BT-like symptoms were recorded from sheep flocks located in Southern Israel, only. In 2018, BTV-3 was detected in sheep, goat and cattle farms situated in southern and central areas of Israel (Figure 1, Tables 3, 4).

The main clinical signs in sheep included pyrexia, nasal discharge, hyperemia and ulceration of the oral and/or nasal mucosa, facial and/or thorax and hindlimbs edema, fatigue, apnea, recumbency, and few abortions. Morbidity in flocks was seen both in young and adult animals and ranged from 5 to 33.3%, with case mortality ranging from 0 to 30% (Table 3). In farms number 1, 2, 6, and 9, no additional pathogen was identified, while in farms number 3, 4, 5, 10, and 11 additional BTV serotypes were contemporaneously identified or/and isolated in the farm or/and in the same animals (BTV-4, 6, 8, Tables 2, 3). In one farm (number 17), where the only clinical sign was abortion, additionally to BTV-3, Shuni virus (SHUV) was detected in plasma sample from an ewe after abortion. Interestingly, in sheep farm from the southern part of Israel, BTV-3 recurrent infection was observed in fall period of 2017 and 2018 (farms number 4 and 9, Tables 3, 4). Moreover, cases of acutely affected animal showing classical BT clinical signs were seen in this farm during 2 month period and confirmed by successful virus isolation (Table 4).

In 2018, BTV-3 was also detected in two goat farms (Tables 3, 4). One was detected in the spleen of a sudden dead goat and the other in a blood sample collected from a doe right after abortion. In the first case, the low BTV-3 load (Ct 32) found in the spleen was the only laboratory finding as neither bacteriological nor toxicological investigation was conducted. Regarding the farm with abortion cases, BTV-4 was also detected in the blood of another doe after abortion. In both farms the pathogen was detected in adult does.

BTV-3 was also identified in field samples collected from 10 cattle farms situated in southern and central areas of the country (Figure 1; Tables 3, 4). Except for the cattle farm number 14, where clinically healthy animals were tested for commercial purposes, BTV-3 was detected in samples of sick and dead cattle received from the beginning of September till the middle of December 2018. In two cattle farms (farms 8 and 15) with unusual mortality rates, *Clostridium perfringens* and *Salmonella* spp. were also found in calves, respectively (Tables 3, 4). In farm number 18, a post parturient BTV-3 positive adult cow was euthanized 2 days after showing hypersalivation, neural ketosis, and neck tense muscle. In addition, a one-year-old calf died after suffering from fever, fatigue, and conjunctival hyperemia. From blood sample of this calf, BEFV and BTV-3 were detected and BTV-3 was successfully isolated. In farm number 20, the clinical cases associated with BTV-3 detection included a calf which died after showing foamy salivation, nystagmus, hypothermia, and mucosal cyanosis and an adult cow showing post parturient ketosis, sharp blindness, endometritis, and tachycardia, which recovered after treatment with antibiotics and anti-inflammatory drugs. BTV-4 was also identified by RT-qPCR in tested samples. Similarly, BTV-3 and BTV-4 mixed infection was detected in a cow with bloody-purulent nasal discharge in farm number 21. Oppositely to previously described cases, no additional pathogens other than BTV-3 were found in farms number 13 and 19. In farm number 13, two adult pregnant cows showed recumbency before dying several days after appearance of clinical signs. No additional laboratory investigations were done for identifying the reason of the death. In farm 19, BTV-3 was detected in an adult cow which recovered after showing fever, indifference, inappetence, and sharp decrease in milk production.

Field samples from ill cattle were not investigated for BTV-15 by RT-qPCR, due to absence of in-house or commercial RT-qPCR validated with currently circulating Israeli strains.

### Sequence, BLAST, Pairwise and Phylogenetic Analysis of the Israeli BTV-3

The three BTV-3 strains isolated in 2013 and 2016 were fully sequenced. Partial sequencing of Seg-2 of the four BTV-3 isolated in 2017 and partial sequencing of Seg-2 of the eight BTV-3 isolated in 2018 revealed very close relationship between strains isolated during the same year. For this reason, two out of four BTV-3 strains isolated in 2017 from two different sheep farms and two out of eight strains isolated in 2018 (one- from sheep from the southern distinct and one from cattle from central distinct of Israel) were selected for further study. The coding regions of all 10 segments of ISR-2019/13, ISR-2153/16, ISR-2262/2/16, ISR-2219/17, ISR-2210/18, and ISR-2255/18 (accession numbers of NCBI GenBank and length of sequenced region of every sequence are shown in Table S2) were almost full sequenced, when most segments of ISR-2396/2/17 strain were sequenced partially. Partial sequences of segment 2 (Seg-2) of all other Israeli BTV-3 isolates were also submitted to NCBI GenBank (accession numbers MH107823, MH107824, MN398282- MN398287) (https://www.ncbi.nlm.nih.gov/nucleotide/).

Israeli BTV-3 strain sequences were also compared and data on nt and aa substitutions were summarized in the Table 5. Due to very close relationship (99.45–99. 92% of nt identity in all genome segments) between BTV-3 strains isolated during 2017–2018 and ISR-2153/16 strain isolated in 2016, only sequences of the ISR-2153/16 strain were considered in the genetic analyses when nt sequences were compared to those of the global BTV strains (Table 6). First Israeli BTV-3 isolate (ISR-2019/13) was used as a prototype Israeli BTV-3 strain. Based on the data on number nt and aa substitutions, as well as on the identity with local or global BTV sequences, genome sequences were considered as homologous, or reassorted (Table 5).

**Table 5:** Comparison between nucleotide (nt) and amino acid (aa) sequences of Israeli BTV-3 strains and ISR-2019/13 strains, which was considered as the prototype Israeli BTV-3 strain.

**Strain**	**ISR-2262/2/16**	**ISR-2153/16**	**ISR-2219/17**	**ISR-2396/2/17**	**ISR-2210/18**	**ISR-2255/18**
Segment	type/nt/aa	type/nt/aa	type/nt/aa	type/nt/aa	type/nt/aa	type/nt/aa
Seg-1	H/16/5	R/220/18	R/215/16	R/NA	R/225/16	R/227/16
Seg-2	H/16/3	H/20/5	H/22/4	H/NA	H/23/4	H/23/4
Seg-3	H/7/0	R/74/1	R/75/0	R/NA	R/75/1	R/76/0
Seg-4^*^	R/36/8	R/105/9	R/107/9	R/108/9	R/107/9	R/106/9
Seg-5	H/5/2	H/6/2	H/6/2	H/NA/NA	H/6/2	H/8/2
Seg-6	H/1/0	H/2/0	H/6/1	H/NA/1	H/10/1	H/11/2
Seg-7	H/3/1	H/4/1	H/4/1	H/NA/NA	H/6/1	H/4/1
Seg-8^*^	H/6/2	H/5/2	H/6/3	H/7/3	H/7/3	H/6/3
Seg-9	R/38/12/0	H/7/2/0	H/8/3/0	^**^H/8/3/0	H/9/4/0	H/9/3/0
Seg-10	R/101/8/16	R/129/12/19	R/131/12/19	R/129/12/19	R/131/14/19	R/131/13/19

* *Segments 4 and 8 of ISR-2019/13 strain are partial. The comparison was done between the same genome regions*.

** *The sequenced region of BTV-3 ISR-2396/2/17 strain is partial and considered from nucleotide position 85. NA, not analyzed. The results were shown in the next sequence: type/-type of origin (homologous (H) or reassorted (R)/nt- number of nucleotide substitutions/ aa- number of amino acid substitutions). In case of segments 9 and 10, where two different open reading frame codding two proteins, aa substitutions in both proteins are shown*.

**Table 6:** Comparison between nt composition of Israeli BTV-3 strains and the closest global BTV strains.

**Strain**	**Segment**	**Closest sequence-nt identity (%)**
ISR−2019/13	Seg-1	KP820917/BTV-2/TUN2000/01/2000-97.71
	Seg-2	MF124293/BTV-3/TUN2016/Zarzis/2016-98.28
	Seg-3	MG255621/BTV-3/ZAF/*O.aries*-tc/ZAF/2017/Smithfield_VR33/2017-97.83
	Seg-4	MF124295/BTV-3/TUN2016/Zarzis/2016-98.93
	Seg-5	JX861492/BTV-1/FRA2007/18^*^-98.19
	Seg-6	AJ586694/BTV-16/NIG1982/10-97.00
	Seg-7	MG255625/BTV-3/*O. aries*-tc/ZAF/2017/Smithfield_VR33/2017-98.17
	Seg-8	KP821732/BTV-1/LIB2007/06-97.61
	Seg-9	KP821904/BTV-24(4)/4ISR2008/02-98.95
	Seg-10	KP821951/BTV-16/CYP2006/01-99.87
ISR−2153/16	Seg-1	JX272389/BTV-22/ZAF/84/184-96.66
	Seg-2	MF124293/BTV-3/TUN2016/Zarzis/2016-98.19
	Seg-4	JN848762/BTV-1/CHN/SZ97/1-95.74
	Seg-5	JX861492/BTV-1/FRA2007/18^*^-98.27
	Seg-6	AJ586694/BTV-16/NIG1982/10-96.75
	Seg-7	MG255625/BTV-3/*O. aries*-tc/ZAF/2017/Smithfield_VR33/2017-97.99
	Seg-8	KP821732/BTV-1/LIB2007/06-97.59
	Seg-9	KP821904/BTV-24(4)/4ISR2008/02-98.76
	Seg-10	MG255688/BTV-12/ZAF/*O.aries*-tc/ZAF/2017/Queenstown_VR55/2017-97.13
ISR−2262/2/16	Seg-1	KP820917/BTV-2/TUN2000/01/2000-97.66
	Seg-2	MF124293/BTV-3/TUN2016/Zarzis/2016−98.19
	Seg-3	MG255621/BTV-3/ZAF/*O.aries*-tc/ZAF/2017/Smithfield_VR33/2017-97.73
	Seg-4	KP821302/BTV-24(4)/ISR2008/02/2008-99.39
	Seg-5	JX861492/BTV-1/FRA2007/18^*^-98.31
	Seg-6	AJ586694/BTV-16/NIG1982/10-96.94
	Seg-7	MG255625/BTV-3/*O*. *aries*-tc/ZAF/2017/Smithfield_VR33/2017-97.93
	Seg-8	KP821732/BTV-1/LIB2007/06-97.44
	Seg-9	KP821870/BTV-2/FRA2001/03-97.13
	Seg-10	FJ713330/BTV-19/600579-92.47

* *Strain sharing the same nt identity with several other BTV strains belonging to BTV-1 and BTV-4*.

#### Seg-1

According to BLAST and pairwise analyses, the ISR-2019/13 and the ISR-2262/2/16 strains were closely related (99.59% nt and 99.61 aa identity, Table 5). They also showed high degree of nt sequence identity with Tunisian (TUN2000/01) and French (FRA2001/01) BTV-2 strains, with which they shared 97.63–97.71% identity, respectively. The ISR-2153/16 strain was instead most closely related to the BTV-22 84/184 strain, with which it shared 96.66% nt identity (Table 6, Figure S1A).

The ISR-2019/13 and the ISR-2262/2/16 strains clustered with BTV-2 and BTV-4 strains isolated in France, Spain and Morocco between 2001 and 2004 while the ISR-2153/16 strain clustered with BTV-16,−22, and−24 African strains.

#### Seg-2

The pairwise analysis showed that all isolated BTV-3 Israeli strains were closely related, sharing 99.39–99.52% nt and 99.47–100% aa identity (Table 5). Israeli BTV-3 strains isolated in 2017 (ISR-2219/17 and ISR-2396/2/17) and 2018 (ISR-2210/18 and ISR-2255/18) were most closely related to the ISR-2153/16 strain. According to the pairwise and BLAST analyses, the Israeli BTV-3 nt sequences showed a very high identity (98.12–98.28%) with the BTV-3 TUN2016/Zarzis strain (Table 6).

**Figure 2:**
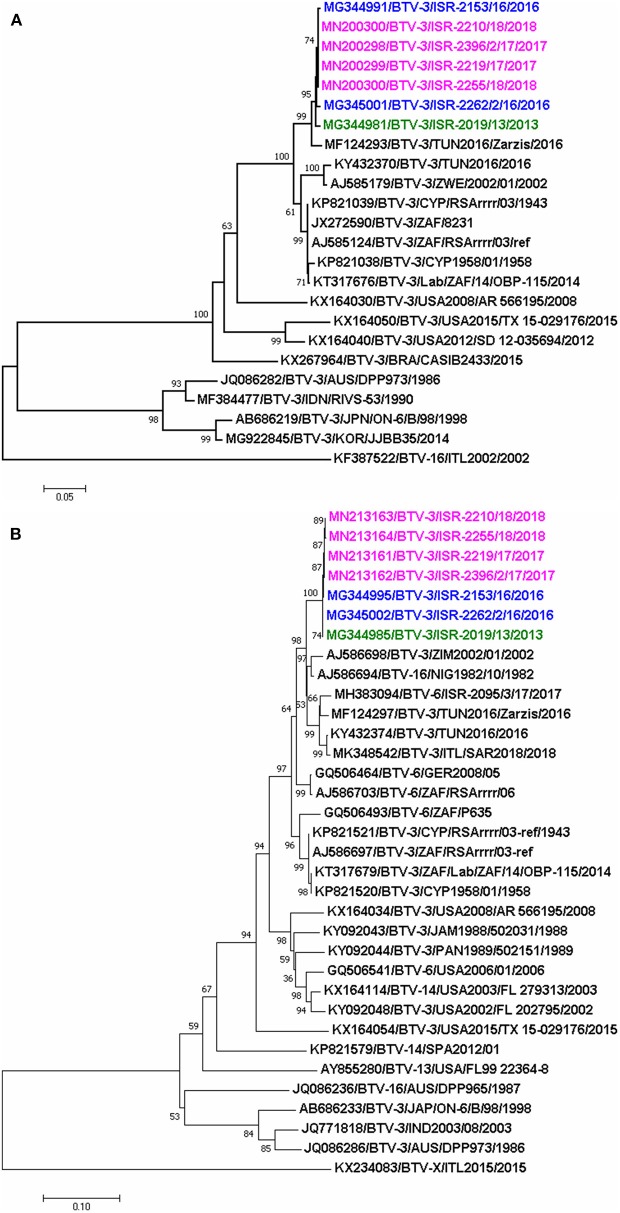
Phylogenetic trees of segment 2 and 6 of Israeli BTV-3 isolates compared with globally published, closely related BTV sequences **(A)** phylogenetic analyses of segment-2; **(B)** phylogenetic analyses of segment 6. Sequences were analyzed and phylogenetic relationships were inferred by using the Neighbor-Joining method. Numbers below branches indicate bootstrap values, based on 1,000 replicates. As outgroup BTV-16 (ITL2002) was used for phylogenetic tree of segment 2, when BTV-X ITL2015 was used as outgroup for phylogenetic tree of segment 6. Israeli BTV-3 strain from 2013 is signed in green, 2016- in blue, and 2017–2018- in pink colors. Viruses are identified by accession number/serotype/isolate/location/year.

The phylogenetic analysis showed that Israeli and Tunisian BTV-3 belonged to the same western topotype/lineage; based on Seg-2 analyses, two different subclusters of closely related BTV-3 isolates circulating in Africa and the Mediterranean region were revealed. One included the closely related BTV-3 Israeli and the BTV-3 TUN-Zarzis/2016 isolates, the second subcluster included BTV-3TUN2016 and the BTV-3 ZIM2002/01 strain from Zimbabwe (Figure 2A).

#### Seg-3

The BLAST and the pairwise analyses showed that the BTV-3 ISR-2019/13 and the ISR-2262/2/16 strains had almost identical nt sequences (99.75% nt and 100% aa identity, Table 5). They showed also high identity (97.73–97.83%) with the South African BTV-3 strain 3/*O. aries*-tc/ZAF/2017/Smithfield_VR33 (Table 6). The ISR-2153/16 and South African BTV-5 *O. aries*-tc/ZAF/2011/Benoni_01012015 strains were most closely related to each other, sharing 98.41% of nt identity (Table 6).

The phylogenetic analysis of Seg-3 nt sequences also revealed two sub-clusters. The ISR-2019/13 and the ISR-2262/2/16 strains grouped with South African BTV-3 and BTV-16 strains isolated in 2017 (3/*O. aries*-tc/ZAF/2017/Smithfield_VR33 and 16/*O. aries*-tc/ZAF/2017/Bethal_VR08 strains, respectively, Figure S1B), while the ISR-2153/16, ISR-2219/17, ISR-2296/2/17, ISR-2210/18, and ISR-2255/18 strains clustered with the South African BTV-4 *O. aries*-tc/ZAF/2017/Smithfield VR30 strains and BTV-5 *aries*-tc/ZAF/2011/Benoni_01012015 strains (Figure S1B).

#### Seg-4

The BLAST and pairwise analyses showed a very high nt sequence identity (98.93%) between the BTV-3 ISR-2019/13 and TUN2016/Zarzis strains. The ISR-2153/16 strain was most closely related to Chinese, South African, and Nigerian BTV-1 and BTV-16 (strains SZ97/1, NIG1982/10, and 5012, respectively), with which it shared 95.39–95.74% nt identity. The ISR-2262/2/16 strain was most closely related to Israeli BTV-24 (the representative strain ISR2008/02), with which it shared 99.39 % nt identity (Table 6, Figure S1C).

Except for the ISR-2153/16 –like isolates, which formed a separate branch, the phylogenetic analysis showed that Tunisian and the other Israeli BTV-3 strains were closely related and formed a single cluster with some BTV-2,−4, and−24 strains that circulated in the Mediterranean Basin between 2000 and 2010. The phylogenetic analysis also confirmed the very high nucleotide sequence identity between the ISR-2019/13 and the TUN-Zarsis/2016 strains, which clustered together, and between the ISR-2262/2/16 and Israeli BTV-24 strains (Figure S1D).

#### Seg-5

The BLAST and the pairwise analyses confirmed that all Israeli BTV-3 strains had high nt and aa sequences (99.64–99.71% and 99.47–100% respectively, Table 5). They also showed high nt (98.19–98.31%) identity with BTV-1 and BTV-4 strains from the Mediterranean Basin (Table 6). The phylogenetic analysis of Seg-5 resulted in a single cluster grouping Israeli BTV-3 strains and BTV-1 and BTV-4 strains circulating in the Mediterranean Basin between 2006 and 2010 (Figure S1E).

#### Seg-6

The pairwise analysis showed that the three Israeli BTV-3 strains were closely related, sharing 99.82–99.94% nt and 99.61–100% aa identity (Table 5). According to the BLAST and the pairwise analyses, sequences of the Israeli strains showed up to 97% identity with the Nigerian BTV-16 NIG1982/10 strain (Table 6, Figure 2B).

Phylogenetic analysis of Seg-6 showed that the Israeli BTV-3 strains formed a separate branch which clustered with Nigerian BTV-16 (NIG1982/10), ZIM2002/01 BTV-3 strain from Zimbabwe, German and Israeli BTV-6 strains (GER2008/05 and ISR-2095/3/17, respectively) and Tunisian BTV-3 TUN2016 and TUN2016/Zarzis strains (Figure 2B).

#### Seg-7

The BLAST and the pairwise analyses showed that Israeli BTV-3 strains had almost identical nt and aa sequences (99.65–99.73% and 99.41–100%, respectively, Table 5). They also showed highest identity with the South African BTV-3 3/*O. aries-tc*/ZAF/2017/Smithfield_VR33 strain (97.93–98.17%) (Table 6). Phylogenetic analysis confirmed the close relationship between Israeli BTV-3 and South African BTV-3 3/*O*. *aries-tc*/ZAF/2017/Smithfield_VR33 strains (Figure S1E).

#### Seg-8

The BLAST and the pairwise analyses also showed a very high nt and aa sequence identity between Israeli BTV-3 strains (99.14–99.82% and 98.85–100%, respectively, Table 5). A high nt identity (97.30–97.61%) was also observed with the Libyan BTV-1 LIB2007/06 strain (Table 6). Phylogenetic analysis showed that Israeli BTV-3 strains formed a separate branch which clustered with Libyan BTV-1 strain LIB2007/06, BTV-2 French and Tunisian strains (TUN2000/01 and FRA2001/03, respectively), BTV-4 SUD1983/01 strain from Sudan and BTV-5 South African strain 5/ZAF/*O.aries-tc/*ZAF/2011/Benoni_01012015 (Figure S1F).

#### Seg-9

The pairwise and the BLAST analyses showed a very high nt and aa sequence identity (99.33–99.39%, respectively) between BTV-3 ISR-2019/13 and ISR-2153/16 strains (Table 5). They also showed high nt sequence identity with the Israeli BTV-24 ISR2008/02 strain (98.76–98.95%, Table 6). Conversely, the BTV-3 ISR-2262/2/16 strain was most closely related to the BTV-2 FRA2001/03 French strain with which it shared 97.13% nt identity (Tables 5, 6).

Phylogenetic analysis showed that Israeli BTV-3 ISR-2019/13 and ISR-2153/16 strains formed a separate sub-cluster with the Israeli BTV-24 2008/02 strain. The ISR-2262/2/16 strain instead clustered with the British BTV-8 8UKG2007/06 strain (Figure S1G).

#### Seg-10

The BLAST and pairwise analysis of Seg-10 nt sequences showed that the BTV-3 ISR-2019/13 strain was closely related to Israeli BTV-5, 16 and 24 (strains ISR-1405/11; ISR-1794/4/12; ISR-3027/6/10, respectively), and to Cypriot BTV-16 CYP2006/01 strain, sharing 99.62–99.87% nt identity (Table 6, the only CYP2006/01 strain is shown). The BTV-3 ISR-2153/16 strain was closely related to some European BTV-8 (UKR2007/06 and NET2006/04 strains), BTV-12 *O.aries-tc*/ZAF/2017/Queenstown_VR55and South African BTV-18 (BT32/76 and 600578 strains), sharing 97.13% nt identity (only BTV-12 12/ZAF/*O.aries*-tc/ZAF/2017/Queenstown_VR55 strain is shown in Table 6). The BTV-3 ISR-2262/2/16 strain had the closest relationship with BTV-19 (600579 and RA 19 OP strains), sharing 91.88–92.47% nt identity (Table 6).

The phylogenetic analysis showed that the ISR-2019/13 strain clustered with local (Israeli and Cypriot) BTV-5,−16, and−24 strains. ISR-2262/2/16 strain clustered with BTV-19 strains, while the ISR-2153/16 strain clustered with South African BTV-12,−14 and 18 (strains 12/ZAF/*O.aries*-tc/ZAF/2017/Queenstown_VR55, BT87/59, and BT32/76, respectively; Figure S1H).

## Discussion

The first recorded cases of BTV-3 in the Mediterranean Basin date back in the middle of 20th century, when BTV-3 was isolated from sheep samples in 1943 and 1958 (www.ncbi.nlm.nih.gov/pubmed). For the next several decades, no evidence of BTV-3 circulation was reported. BTV-3 circulation has been recently reported in Tunisia, Egypt and Italy. Even though detected either in goats or cattle (7, 38, 54) in these countries clinical signs associated to BTV-3 infection were observed and described in sheep only (7, 38, 39). According to the retrospective findings of this study, during 2013–2017 BTV-3 outbreaks were restricted to Negev Desert area, in the southern district, while in 2018 it spread among most coastal and some central areas of Israel (Figure 1, Table 3). An additional difference between 2018 and 2013–2017 BTV-3 outbreaks was the exposure of Israeli goats and cattle in 2018 outbreaks. In this year, in fact, BTV-3 infection was occasionally associated with BT typical and atypical clinical signs, occurrence which was never observed before either in Israel or in the Mediterranean region (7, 38, 54). These events may imply an increased infectivity and pathogenicity of the BTV-3 Israeli strains circulating in 2018 or/and their adaptation to the local vectors. However, because of the exiguous number of samples from diseased and clinically healthy animals received from the BTV-3 affected farms, it was hard to estimate the real exposure of ruminants and real effect BTV-3 infection on these animals. In Israeli goats and cattle, making any conclusion regarding the role of BTV-3 in causing illness and death was even harder due to involvement of different bacterial and viral agents in the clinical cases or to the absence of bacterial and toxicological investigations in some fatal cases. According to what observed in this survey, all Israeli BTV-3 strains were definitely capable of inducing classical manifestations of BT disease in sheep. These symptoms were more severe in case of BTV mixed infections (BTV-3 and BTV-4, BTV-6, or BTV-8). Notably, cases of acutely affected animal showing classical BT clinical signs were seen in one sheep farm during two-month period and confirmed by successful virus isolation. This fact illustrated presence of newly infected animals and probably infected vector during prolonged period during two seasons 2017 and 2018 (farms number 4 and 9, Table 4).

Interestingly, in some cattle and sheep farms, BTV-3 as well as BTV-4 were detected in fetal tissues, placenta and in newborn animals. Although these findings were not sufficient to establish the definitive involvement of BTV-3 in determining reproductive failures, its presence in fetal tissues and/or newborn animals, provided evidence of its capability to infect placenta and probably also cross the placental barrier. As far as we know, apart from BTV-8, causing numerous abortions and malformations, and lastly BTV-1, this capability is proper of vaccine or lab derived strains (18, 55).

Higher susceptibility observed in sheep reflected the number of successful virus isolations achieved in this species (33%) compared to cattle (7.7%), which, in turn, may imply a higher number of sheep acutely infected by BTV-3 than cattle.

Two different strains named TUN2016 and TUN2016/Zarzis have been identified as responsible of the Northern African and Italian BTV-3 outbreaks (7, 38, 39, 54). This study revealed that three additional BTV-3 strains have been circulating in the Mediterranean Basin and in Israel, in particular, at least since 2013. Genome comparison allowed a tentative reconstruction of the ancestral viral genome of these strains. When the Israeli BTV-3 strain sequences were compared, it was evident that Seg-2, 5, 6, 7 and 8 of the Israeli strains derived from a common ancestor. According to Seg-2 phylogenetic analyses, the BTV-3 Israeli strains also shared common ancestors with the Tunisian TUN2016/Zarzis strain. For the ISR-2019/13 strain, this was evident for Seg-4 too.

In all these strains, reassortment phenomena were also found. When compared with the prototype ISR-2019/13 strain, the ISR-2262/2/16 and the ISR-2153/16-like strains have 3 and 4 reassorted segments, respectively. Moreover, their Seg-4, Seg-9, and Seg-10 sequences clearly evidenced a different origin. “Traces” of untyped South African strains and BTV serotypes (BTV-18, BTV-19, BTV-22) exotic for the region in the Israeli BTV-3 genomes were clear, at least in their last segments. These results may indicate the circulation of exotic strain/serotypes in the region.

Between 2013 and 2017, only a low proportion of the BTV strains isolated from sick domestic ruminants was identified as BTV-3. Thus, in 2013 only one out of 15, in 2016, 3 out of 58, in 2017 4 out of 40 virus isolates were BTV-3. In these years, the BTV-3 circulation was limited to southern Israel only. In 2018, the number of BTV-3 isolates among the total number of BTV isolated, sharply increased (8 out of 25). The BTV-3 circulation also spread along all coastal area of Israel, suggesting an increased infectivity of the BTV-3 ISR-2153/16 strain among susceptible Israeli domestic ruminants which can be explained by point mutations both in coding and non-coding regions (not shown in this work) or possible introduction closely related viruses to ISR-2153/16 strain into Israel.

In spite of a long history of BTV-3 infections in ruminants in South Africa, India, Caribbean and Northern America (41, 42, 56, 57), epidemiological data as well as information on pathogenicity and infectivity of BTV-3 infection from these regions are absent or scarce. Even if it was not possible to evaluate their pathogenicity in cattle and goats, this paper still gives important information on the possible origin of the BTV-3 strains circulating in the Mediterranean basin, elucidating their possible routes of incursion. However, further investigation is needed to improve our understanding on their biological properties and their impact on livestock.

## Data Availability Statement

The data analyzed in this study can be found in the article/Supplementary Material.

## Ethics Statement

This study did not involve any human participants and animals. Ethical approval was not required for this study according to national legislation.

## Author Contributions

GK, JV, IS, SS, AM, ID, IG, AK, OK, and EO collected field samples and epidemiological data. NG, DD, and VB performed diagnostic tests of field samples. NG developed in-house PCR systems, performed PCR tests for sequencing, analyzed and summarized data of sequencing, and phylogenetic analyses. VB, AG, and NG performed isolations of the viruses in eggs and tissue cultures. AE and AL reviewed the manuscript. NG and GS drafted the manuscript, while VB, AE, and YK discussed the results and commented on the manuscript.

### Conflict of Interest

NG was employed by Kimron Veterinary Institute, Israel. The remaining authors declare that the research was conducted in the absence of any commercial or financial relationships that could be construed as a potential conflict of interest.

## Supplementary Material

Figure S1 Phylogenetic trees of segment 1, 3–5, and 7–10 Israeli BTV-3 isolates compared with globally published, closely related BTV sequences. **(A)** phylogenetic analyses of segment 1; **(B)** phylogenetic analyses of segment 3; **(C)** phylogenetic analyses of segment 4; **(D)** phylogenetic analyses of segment 5; **(E)** phylogenetic analyses of segment 7; **(F)** phylogenetic analyses of segment 8; **(G)** phylogenetic analyses of segment 9; **(H)** phylogenetic analyses of segment 10. Sequences were analyzed and phylogenetic relationships were inferred by using the Neighbor-Joining method. Numbers below branches indicate bootstrap values, based on 1,000 replicates. As outgroup BTV-26 (KUW2010) was used for phylogenetic trees of most segments. Israeli BTV-3 strain from 2013 is signed in green, 2016- in blue, and 2017–2018- in pink colors. Viruses are identified by accession number/serotype/isolate/location/year.

Table S1 List of segment 2 primers used for identification and sequencing of circulating Israeli bluetongue serotypes.

Table S2 Accession numbers of Israeli BTV-3 and sequenced regions.
