# Supplementary figures and images for: Bluetongue Serotype 3 in Israel 2013–2018: Clinical Manifestations of the Disease and Molecular Characterization of Israeli Strains
Source: Front Vet Sci. 2020 Mar 6;7:112. doi: 10.3389/fvets.2020.00112 (PMC7068852; doi:10.3389/fvets.2020.00112)

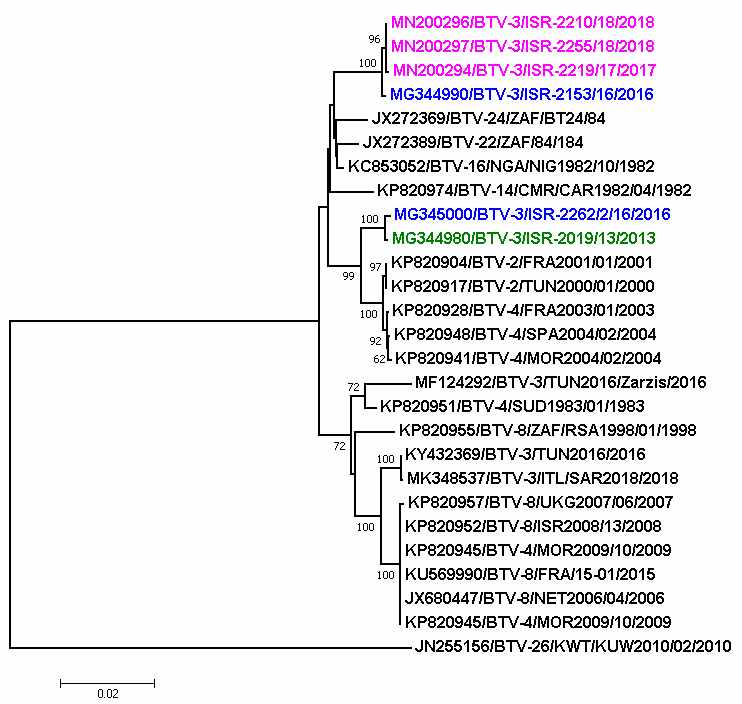

Supplement: Supplementary file 1 [file Image_1.TIFF]

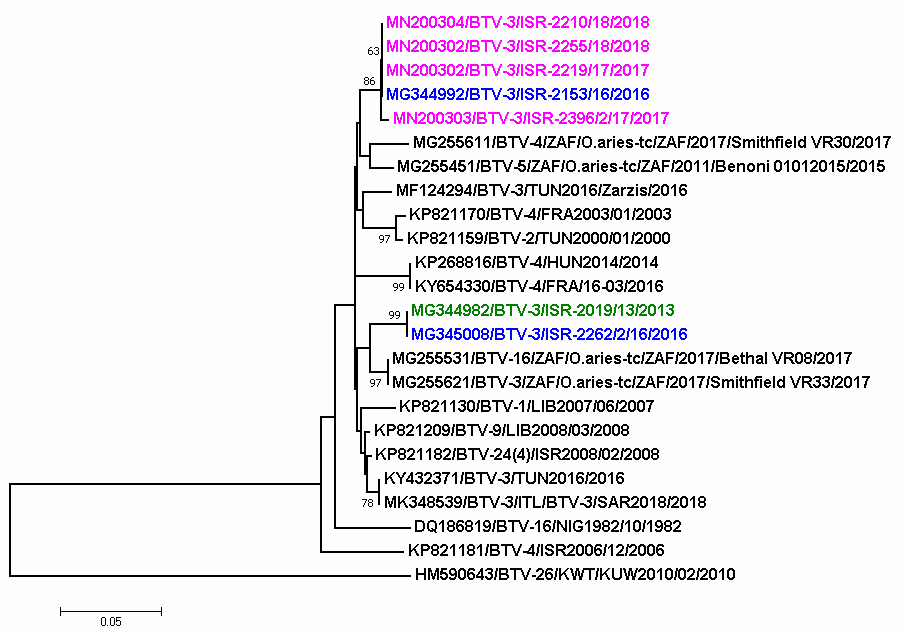

Supplement: Supplementary file 2 [file Image_2.TIFF]

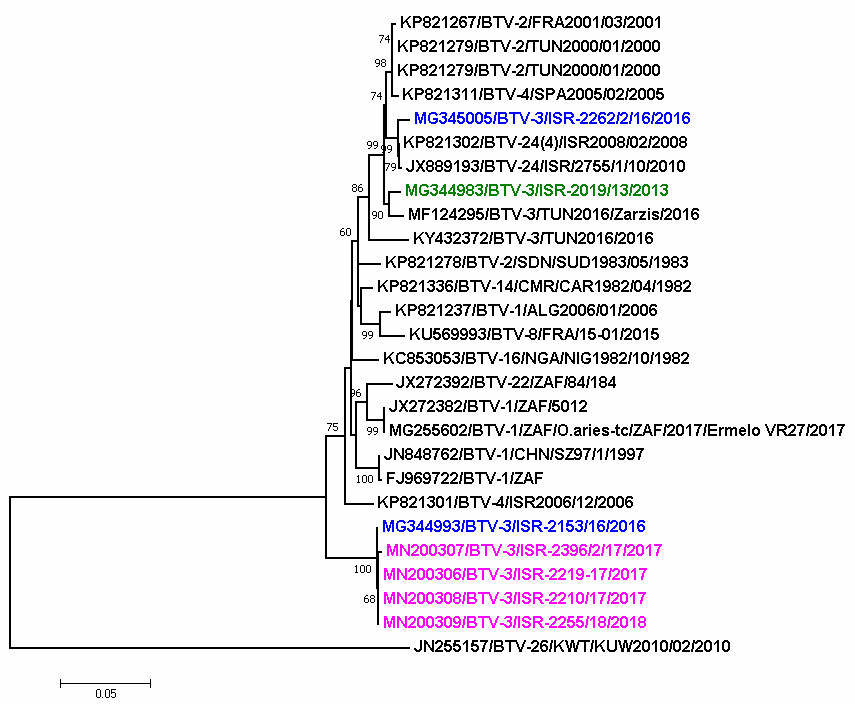

Supplement: Supplementary file 3 [file Image_3.TIFF]

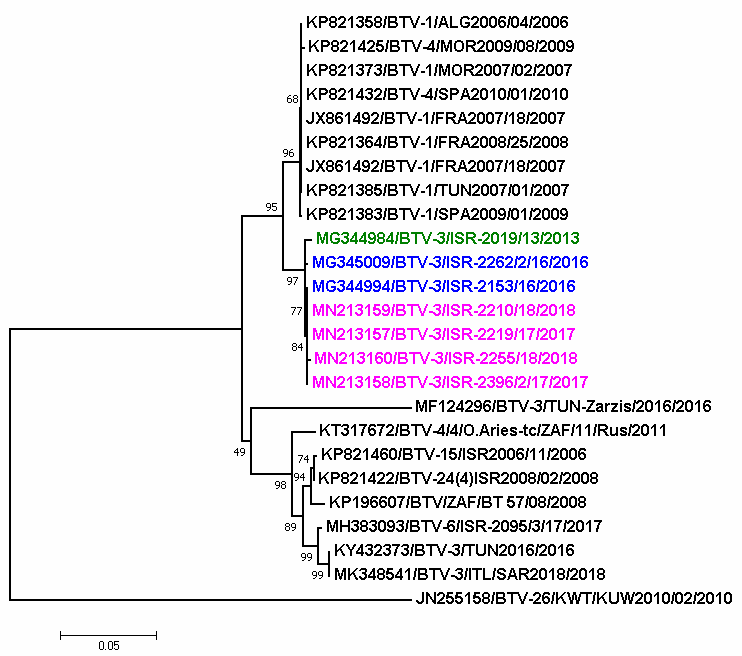

Supplement: Supplementary file 4 [file Image_4.TIFF]

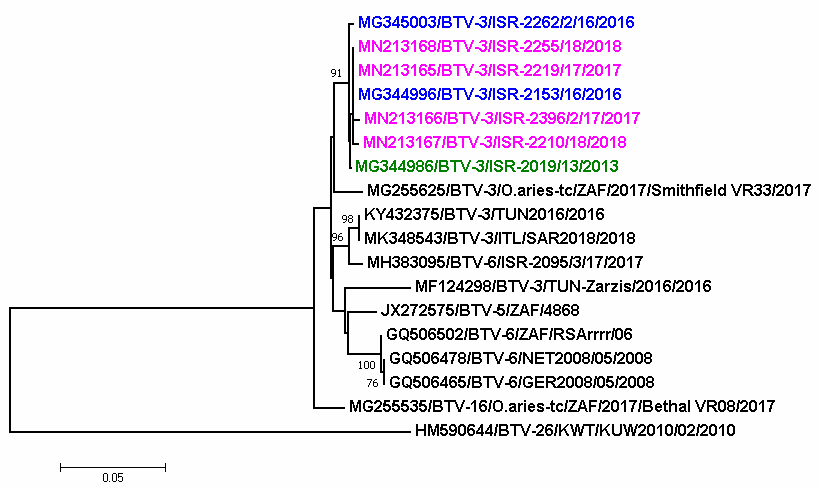

Supplement: Supplementary file 5 [file Image_5.TIFF]

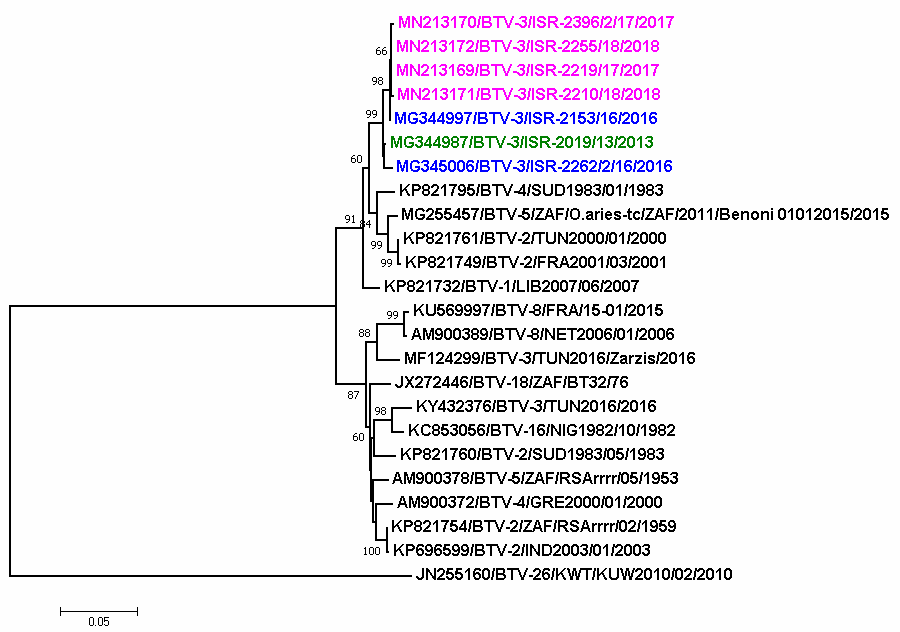

Supplement: Supplementary file 6 [file Image_6.TIFF]

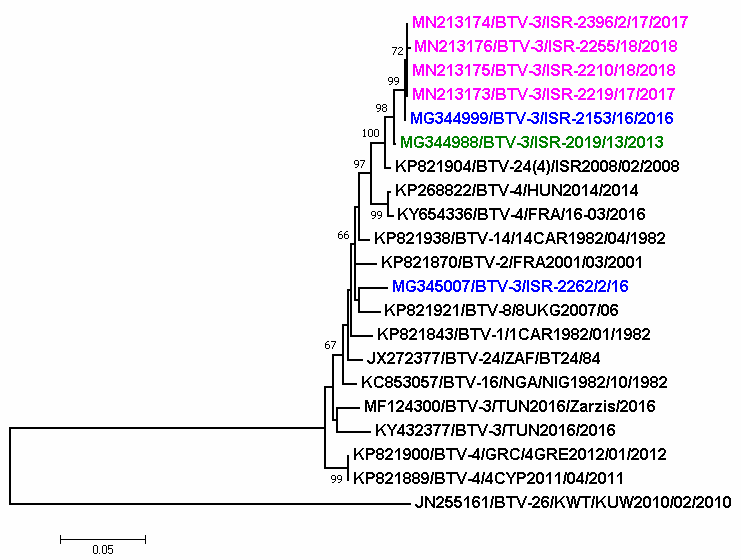

Supplement: Supplementary file 7 [file Image_7.TIFF]

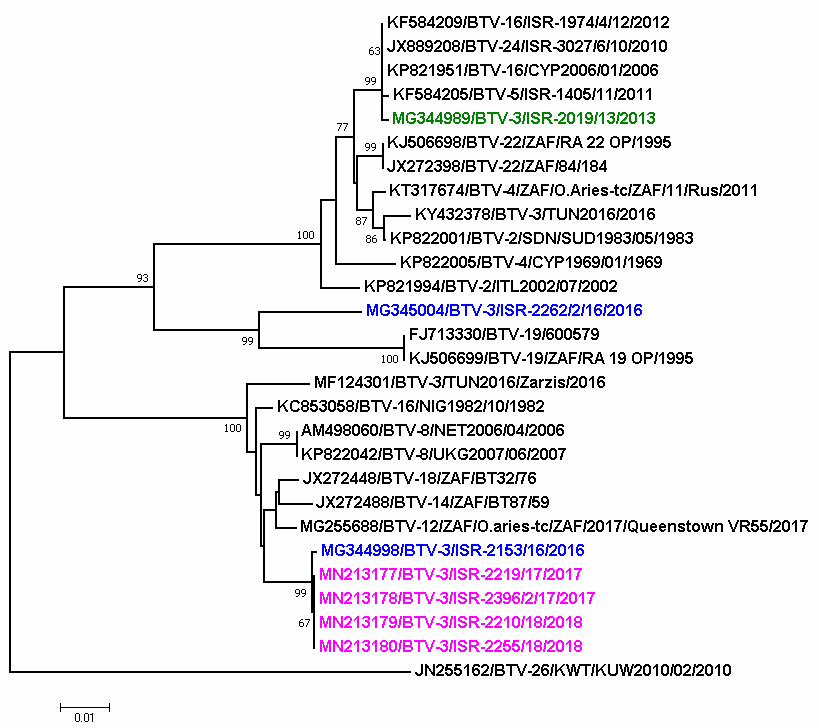

Supplement: Figure S1 — Phylogenetic trees of segment 1, 3–5, and 7–10 Israeli BTV-3 isolates compared with globally published, closely related BTV sequences. (A) phylogenetic analyses of segment 1; (B) phylogenetic analyses of segment 3; (C) phylogenetic analyses of segment 4; (D) phylogenetic analyses of segment 5; (E) phylogenetic analyses of segment 7; (F) phylogenetic analyses of segment 8; (G) phylogenetic analyses of segment 9; (H) phylogenetic analyses of segment 10. Sequences were analyzed and phylogenetic relationships were inferred by using the Neighbor-Joining method. Numbers below branches indicate bootstrap values, based on 1,000 replicates. As outgroup BTV-26 (KUW2010) was used for phylogenetic trees of most segments. Israeli BTV-3 strain from 2013 is signed in green, 2016- in blue, and 2017–2018- in pink colors. Viruses are identified by accession number/serotype/isolate/location/year. [file Image_8.TIFF]
