# Supplementary material for: Bluetongue Serotype 3 in Israel 2013–2018: Clinical Manifestations of the Disease and Molecular Characterization of Israeli Strains
Source: Front Vet Sci. 2020 Mar 6;7:112. doi: 10.3389/fvets.2020.00112 (PMC7068852; doi:10.3389/fvets.2020.00112)
Supplement: Table S1 — List of segment 2 primers used for identification and sequencing of circulating Israeli bluetongue serotypes. [file Table_1.DOCX]

**Table S1**. List of segment 2 primers used for identification and sequencing of circulating Israeli bluetongue serotypes

| serotype | name | sequence | length of product | annealing t°C | source |
| --- | --- | --- | --- | --- | --- |
| 2 | 2VP2-1f | GTTAAAACAGGATCGCGA | 358 | 54 | this study |
|  | 2VP2-340R | CGGCTGTTGATCCATACT |  |  |  |
| 3 | 3VP2-1F | GTT AAA AAC GCT GTC CCG AGA | 658 | 55.5 | this study |
|  | 3VP2-637R | GAG CGC CCA CTC TAA ATT CCT C |  |  |  |
| 4 | VP2-S4-1F | TGTTCCCAAACTAGACATCGTTAG | 493 | 54 | this study |
|  | VP2-4-1R | TACCCATTTCTCCGAACCTA |  |  |  |
| 4 and 24* | BT4-10-24-1F | ATG GAG GAR TTY GTC ATW CCW GT | 329 | 53.4 | this study |
|  | BT4-10-24-330R | TCR TCR ATR GCY CAY TTC ATC CA | 332 |  |  |
| 5 and 9 | BT-5+9-VP2-F | TAT GCR TTG CCA ATY AGY TT | 340 | 51.4 | this study |
|  | BT-5+9-VP2-R | TGT AWG CTC YAG GAG TCY CA |  |  |  |
| 6 | 6VP2-124F | TGTAACCCAAATTCCCACGAA | 923 | 52.6 | Golender et al., 2019 |
|  | 6VP2-1030R | CAGAGGCGGCTATCATA |  |  |  |
| 8 | 8P2-34F | TTC CGA TTT ATA CGA ATG TAT TCC C | 762 | 54 | this study |
|  | 8P2-772R | TCA AAG CGG TTA TTT CCT CTT GTA |  |  |  |
| 12 | 12VP2-1200F | TCG GAA AGA GAT TGA TCG TGA A | 782 | 54.3 | this study |
|  | 12VP2-1962R-C | TGA ACT GAT CCC ACG TAC GA |  |  |  |
| 15 and untyped 57/08 strain | 15-like-VP2-2f | TTA AAA GTT GCM GGG ATG GRA G | 632 | 55.1 | this study |
|  | 15-like-VP2-612R | CCK ATM GCC TTA ATT TTR TAC G |  |  |  |
| 16 | 16VP2-68-F | AACTRGTTGGGCGTTACGAT | 729 | 54 | this study |
|  | 16VP2-777-R | CTAAAGCGGCCATTTCCTCT |  |  |  |
