# Supplementary material for: Bluetongue Serotype 3 in Israel 2013–2018: Clinical Manifestations of the Disease and Molecular Characterization of Israeli Strains
Source: Front Vet Sci. 2020 Mar 6;7:112. doi: 10.3389/fvets.2020.00112 (PMC7068852; doi:10.3389/fvets.2020.00112)
Supplement: Table S2 — Accession numbers of Israeli BTV-3 and sequenced regions. [file Table_2.docx]

**Table S2. Accession numbers of Israeli BTV-3 and sequenced regions**

| segment | 1 | 2 | 3 | 4 | 5 | 6 | 7 | 8 | 9 | 10 |
| --- | --- | --- | --- | --- | --- | --- | --- | --- | --- | --- |
| strain |  |  |  |  |  |  |  |  |  |  |
| ISR-2019/13 | MG344980 | MG344981 | MG344982 | MG344983 | MG344984 | MG344985 | MG344986 | MG344987 | MG344988 | MG344989 |
|  | 3944/1-3928 | 2935/1-2857 | 2772/2-2772 | 1981/190-1971 | 1774/11-1722 | 1637/2-1635 | 1156/1-1147 | 1125/55-1090 | 1050/1-1050 | 822/1-802 |
| ISR-2153/16 | MG344990 | MG344991 | MG344992 | MG344993 | MG344994 | MG344995 | MG344996 | MG344997 | MG344999 | MG344998 |
|  | 3944/1-3927 | 2935/1-2935 | 2772/1-2772 | 1981/1-1974 | 1774/35-1773 | 1637/1-1635 | 1156/1-1147 | 1125/11-1191 | 1050/1-1050 | 822/1-801 |
| ISR-2262/2/16 | MG345000 | MG345001 | MG345008 | MG345005 | MG345009 | MG345002 | MG345003 | MG345006 | MG345007 | MG345004 |
|  | 3944/1-3928 | 2935/1-2935 | 2772/1-2772 | 1981/1-1972 | 1774/4-1773 | 1637/2-1635 | 1156/37-1147 | 1125/35-1090 | 1050/40-1050 | 822/1-822 |
| ISR-2219/17 | MN200294 | MN200298 | MN200302 | MN200306 | MN213157 | MN213161 | MN213165 | MN213169 | MN213173 | MN213177 |
|  | 3944/9-3918 | 2935/1-2923 | 2772/11-2769 | 1981/1-1981 | 1774/2-1765 | 1637/4-1635 | 1156/1-1156 | 1125/1-1125 | 1050/1-978 | 822/1-821 |
| ISR-2396/2/17 | MN200295 | MN200299 | MN200303 | MN200307 | MN213158 | MN213162 | MN213166 | MN213170 | MN213174 | MN213178 |
|  | 3944/11-816 | 2935/1-982 | 2772/109-991 | 1981/39-1948 | 1774/76-1292 | 1637/36-1587 | 1156/27-1050 | 1125/54-1011 | 1050/84-978 | 822/1-802 |
| ISR-2210/18 | MN200296 | MN200300 | MN200304 | MN200308 | MN213159 | MN213163 | MN213167 | MN213171 | MN213175 | MN213179 |
|  | 3944/4-3927 | 2935/1-2928 | 2772/104-2762 | 1981/1-1981 | 1774/11-1773 | 1637/1-1634 | 1156/1-1156 | 1125/1-1125 | 1050/4-1033 | 822/1-790 |
| ISR-2255/18 | MN200297 | MN200301 | MN200305 | MN200309 | MN213160 | MN213164 | MN213168 | MN213172 | MN213176 | MN213180 |
|  | 3944/10-3927 | 2935/1-2928 | 2772/1-2772 | 1981/1-1981 | 1774/11-1773 | 1637/1-1634 | 1156/1-1120 | 1125/1-1125 | 1050/1-1041 | 822/1-802 |

Upper row- accession number; lower low- total length of the segment/sequenced region
